# Comparing methods for risk prediction of multicategory outcomes: dichotomized logistic regression vs. multinominal logit regression

**DOI:** 10.21203/rs.3.rs-3911212/v1

**Published:** 2024-02-05

**Authors:** lei li, Matthew A. Rysavy, Georgiy Bobashev, Abhik Das

**Affiliations:** RTI Intenational; University of Texas Health Science Center at Houston; RTI International; RTI International

**Keywords:** Risk prediction, Multicategory outcome, Discrimination, Calibration, Competing risks, Preterm infant (limit: 3–10)

## Abstract

**Background:**

Medical outcomes of interest to clinicians may have multiple categories. Researchers face several options for risk prediction of such outcomes, including dichotomized logistic regression and multinomial logit regression modeling. We aimed to compare these methods and provide practical guidance needed.

**Methods:**

We described dichotomized logistic regression and competing risks regression, and an alternative to standard multinomial logit regression, continuation-ratio logit regression for ordinal outcomes. We then applied these methods to develop prediction models of survival and growth outcomes based on the NICHD Extremely Preterm Birth Outcome Tool model. The statistical and practical advantages and flaws of these methods were examined and both discrimination and calibration of the estimated models were assessed.

**Results:**

The dichotomized logistic models and multinomial continuation-ratio logit model had similar discrimination and calibration in predicting death and survival without neurodevelopmental impairment. But the continuation-ratio logit model had better discrimination and calibration in predicting probabilities of neurodevelopmental impairment. The sum of predicted probabilities of outcome categories from the logistic models did not equal 100% for about half of the study infants, ranging from 87.7% to 124.0%, and the logistic model of neurodevelopmental impairment greatly overpredicted the risk among low-risk infants and underpredicted among high-risk infants.

**Conclusions:**

Estimating multiple logistic regression models of dichotomized outcomes may result in poorly calibrated predictions. For an outcome with multiple ordinal categories, continuation-ratio logit regression is a useful alternative to standard multinomial logit regression. It produces better calibrated predictions and has the advantages of simplicity in model interpretation and flexibility to include outcome category-specific predictors and random-effect terms for patient heterogeneity by hospital.

## Background

Multivariable risk prediction models are routinely used by healthcare providers in patient counseling and clinical decision-making. The outcomes of these models are often binary and the algorithm is typically based on logistic regression. While outcomes of many medical conditions can have more than two categories, they may be dichotomized by combining multiple categories together and modeled using logistic regression. For an outcome of death, illness or illness-free survival, for example, a single category, illness-free survival, or a combined category, death or illness, may be of interest and modeled. Although multinomial logit models can simultaneously predict probabilities of multiple outcome categories and thus have the advantage of avoiding loss of detailed information, they are not known to have superior predictive performance.

Few studies have compared predictive performance of logistic models and multinomial logit models. Biesheuvel et al. and Roukema et al. assessed model discrimination and did not find a meaningful difference [1, 2]. More recently, Van Calster and McLernon et. al. argued that model calibration performance should not be overlooked and poor calibration may make a prediction model clinically useless or even harmful [3]. A study by Edlinger et al. focused on calibration performance of alternative multinomial models for ordinal outcomes but did not compare with that of logistic models of dichotomized outcomes [4].

In this paper, we describe alternative methods for modeling multicategory outcomes. Using data on mortality and neurological development among extremely preterm infants, we develop logistic and multinomial logit risk prediction models and assess both model discrimination and calibration. We also compare their statistical advantages and flaws, and differences in model interpretation.

## Methods

We consider risk prediction for a multicategory outcome among patients admitted into a variety of hospitals. Let $Y_{si}$ indicate an outcome with J categories of the ith patient in the sth hospital, and $X_{si1}-X_{si5}$, for instance, be five predictor variables selected for inclusion in the model. With data collected on patients in multiple hospitals, patient heterogeneity by hospital can cause poor predictive performance [4, 5]. We try to add hospital random-effect terms in our models to account for hospital-level variation in outcomes.

### Dichotomized logistic regression

Let the probability of outcome category j be $\pi_{si}(j)=Pr\left\{Y_{si}=j\right\}$. A logistic regression model can be estimated for each outcome category,

$$logit\left(\pi_{si}(j)\right)=\beta_{0j}+\beta_{1j}X_{si1}+\beta_{2j}X_{si2}+\beta_{3j}X_{si3}+\beta_{4j}X_{si4}+\beta_{5j}X_{si5}+a_{s},j=1,\ldots ,J$$

where the intercept $b_{0j}$ and coefficients $b_{1j}-b_{5j}$ are parameters to be estimated. We further assume that the hospital random-effect term $a_{s}$ follows a Normal distribution with zero mean and a constant variance. A drawback of this method is that sum of predicted probabilities over all outcome categories for a patient is not constrained to 100%.

### Multinomial continuation-ratio logit regression

As an extension of logistic regression, a standard multinomial logit model simultaneously fits J-1 logit models of each outcome category relative to a fixed reference category and constrains the sum of all predicted probabilities to 100%, that is, $\sum_{j=1}^{J}\;\pi_{si}(j)=1$. A limitation of this method is that the use of a same reference category and the inclusion of random-effect terms can make model estimation and interpretation difficult [6].

For an outcome with ordered categories, it is preferable to use alternative forms of multinomial logit models that exploit ordinal nature of the outcome categories [7]. We model the sequentially defined conditional probability in the jth category or higher, $\pi_{si}\left(j|Y_{si}\geq j\right)=Pr\left\{Y_{si}=j|Y_{si}\geq j\right\}$. The continuation-ratio logit models are of the following form,

$$Logit\left(\pi_{si}\left(j|Y_{si}\geq j\right)\right)=\beta_{0j}+\beta_{1j}X_{si1}+\beta_{2j}X_{si2}+\beta_{3j}X_{si3}+\beta_{4j}X_{si4}+\beta_{5j}X_{si5}+a_{si},\;\;j=1,\ldots ,J-1$$

We also assume that the random-effect terms $\left(a_{s1},\ldots ,a_{s(J-1)}\right)$ jointly follow a multivariate Normal distribution with zero means [8]. Various forms of the variance-covariance matrix may be specified to represent the correlation structure among the continuation-ratio logits. A simple diagonal form, for example, indicates independent random effects.

### Logistic competing risks regression

Competing-risk bias is often a concern in dichotomized logistic regression estimation and to overcome this bias composite outcomes combining competing-risk categories such as illness or death are commonly used as study endpoints [9, 10]. A statistical method developed to model time-to-event data adjusting for competing risks, logistic competing risks regression, can be potentially useful [11]. Let $T_{\mathrm{sij}}$ be time to the occurrence of event j of patient i in hospital s, the cumulative incidence function by a preset time t for an event of interest, say j=1, is then defined as $F_{si1}(t)=Pr\left\{T_{si1}<=t\right\}$. The logistic competing risks regression fits a model of binary outcome of the occurrence of the event by time t,

$$logit\left\{F_{sij}(t)\right\}=\beta_{0j}(t)+\beta_{1j}X_{si1}+\beta_{2j}X_{si2}+\beta_{3j}X_{si3}+\beta_{4j}X_{si4}+\beta_{5j}X_{si5}$$

A nice feature of this model is that the coefficients can be interpreted in terms of odd ratios.

## Results

### Patient outcomes and predictor variables

We obtained data on 3927 infants who were born extremely preterm in 19 hospitals in the U.S. and enrolled at birth into an observational study [12]. These infants did not have major congenital anomalies and received postnatal intensive care. All the surviving infants completed assessments of neurodevelopmental impairment (NDI) at a single timepoint of 22–26 months’ age corrected for prematurity [13]. NDI is a comprehensive measure of child development based on structured physical examinations and functional assessments. Informed consents were obtained for all infants at hospitals that required parental consent.

For simplicity, we created an outcome with three ordered categories, death, survival with NDI, or survival without NDI (NDI-free survival), and selected five predictor variables, birth weight and gestational age, sex, singleton birth, and exposure to antenatal corticosteroids. These variables have been previously included in the widely used NICHD Extremely Preterm Birth Outcome Tool model [14, 15]. The birth weights and gestational ages of the infants ranged from 401 to 1000 grams (mean: 675 grams) and 22 to 25 weeks (22–23 weeks: 21%), 47% were female, 74% were singleton births, and 85% received antenatal corticosteroids.

### Estimated models

We used SAS PROC GLIMMIX to fit random-effect logistic models and continuation-ratio logit model and the R package *riskRegression* to fit logistic competing risks model [11, 16]. We should note that the original patient-level data file should be re-structured such that a patient could have as many as J–1 records stacked together for the estimation of continuation-ratio logit model, and age in days at death or date of NDI examination was used as time to event of interest for the estimation of logistic competing risks model.

The estimated odds ratios of the predictor variables and the variances of the random hospital effects from three separate logistic models of dichotomized outcomes, death (vs survival), NDI (vs death or NDI-free survival) and NDI-free survival (vs death or NDI) and a multinomial continuation-ratio logit model that jointly predicts the probabilities of death (vs survival) and NDI (vs NDI-free survival) are presented in Table 1. The predictor variables showed similar effects on death but very different effects on NDI. Notably, antenatal corticosteroid exposure had a significant and positive effect on NDI in the logistic model and a significant but negative effect on NDI in the continuation-ratio logit model. Also, the large variance estimates of the random hospital effects relative to their standard errors in these models suggested significant differences in outcomes among hospitals.

Considering NDI an outcome category of interest and death a competing risk with NDI, we estimated a logistic competing risks regression model of NDI and a logistic model of composite outcome of NDI or death. The estimation results are presented in Table 2. We can see that the odds ratios of the predictor variables from the logistic competing risk model were quite close to those from the logistic model of NDI and the odds ratios from the logistic model of NDI or death were quite close to those from the logistic model of death.

### Model predictive performance

We computed the Brier scores and C-statistics to assess discrimination and general validity. To correct for statistical optimum we generated 200 bootstrap samples drawn with replacement from the model predicted probabilities [17, 18]. Four increasingly stringent levels of calibration have been suggested for measuring model calibration, mean, weak, moderate, or strong calibration [3]. We assessed model calibration at the first three levels using means and ranges of predicted probabilities, calibration intercepts and slopes and calibration plots.

Measures of predictive performance of the logistic models and the continuation-ratio logit model are summarized in Table 3. The similar Brier scores and C-statistics indicate similar overall model validity and discrimination. The large C-statistics (> 0.7) for death and NDI-free survival suggest equally satisfactory discrimination, but the lower and slightly different C-statistics for NDI, 0.623 for the logistic model and 0.637 for the continuation-ratio logit model, suggest less satisfactory discrimination. The means of the predicted probabilities of death, NDI and NDI-free survival also are nearly same, indicating similar calibration. But the predicted probabilities of NDI from the logistic model had a slightly narrower range (8.5% – 48.8% vs. 6.6% – 52.1%). A more notable difference, however, is that the sum of all predicted probabilities from the logistic models did not equal 100% for about half of all study infants, ranging from 87.7–124.0%, but the sum from the continuation-ratio logit model equaled 100% for all study infants. The calibration intercepts and slopes were similarly close to zero and one for death and NDI-free survival, but slightly greater than zero and one for NDI.

**Figure 1:**
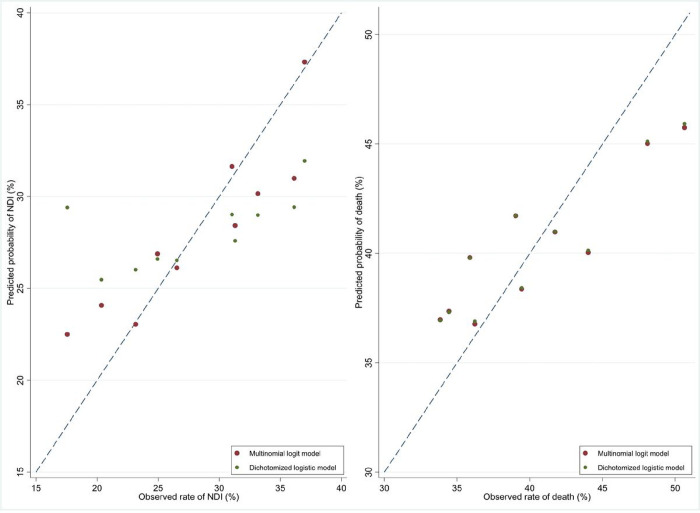
Mean predicted probabilities of neurodevelopmental impairment from dichotomized logistic models and multinomial continuation-ratio logit model and observed rates

**Figure 2:**
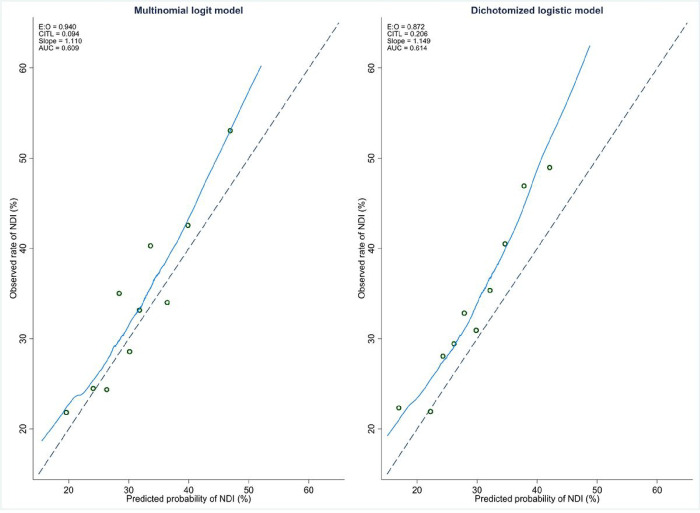
Calibration plots of predicted probabilities of neurodevelopmental impairment</P/>E:O - Ratio of predicted to observed, CITL - Calibration-in-the-large, Slope - Calibration slope, AUC - C-statistic

We further assessed model calibration by exploiting the fact that the predicted probabilities for each patient from the logistic regression models did not add up to 100%. We divided all the infants into decile groups by the sums of their predicted probabilities and calculated the means of the model predicted probabilities. In Fig. 1, we can see that the means of the predicted probabilities of NDI from the continuation-ratio logit model tended to track the observed rates more closely. But those from the logistic model were much higher than the observed rates at the lower end of the observed rates and much lower than the observed rates at the higher end of the observed rates. We noted that infants in the three groups with the lowest observed rates had sums of the predicted probabilities greater than 100% and infants in the three groups with the highest observed rates had sums of the predicted probabilities less than 100%. The mean predicted probabilities of death from the continuation-ratio logit model and those from the logistic model nearly overlapped. They agreed well with the observed rates. We also compared calibration plots of the predicted probabilities among infants whose sums of the predicted probabilities did not equal 100% in Fig. 2. The predicted probabilities of NDI from the logistic model had a smaller ratio of the predicted to the observed (0.872 vs 0.940) and a larger calibration intercept (0.206 vs 0.094).

Because the estimated competing risks model had odds ratios close to those from the logistic model of NDI and the estimated logistic model of the composite outcome of NDI or death had odds ratios that were the inverse of the logistic model of NDI-free survival, they should also have similar discrimination and calibration. Additionally, we computed C-statistics of the logistic model of the composite outcome for predicting death or NDI alone. Prediction of death (AUC=.719) was moderate, but prediction of NDI was poor (AUC=.485).

## Discussion

Risk prediction models are important tools in clinical decision-making and prognosis often takes the form of multiple categories. We have compared two commonly used methods for modeling multicategory outcomes, dichotomized logistic regression and multinomial logit regression, in an application of predicting mortality and neurodevelopmental impairment among extremely preterm infants. Because the outcome has three ordinal categories, we also used an alternative multinomial logit model, continuation-ratio logit model.

We assessed both discrimination and calibration of the estimated models. Consistent with the findings by Biesheuvel et al. and Roukema et al. [1, 2], our results showed that the logistic models and continuation-ratio logit models had similarly satisfactory discrimination in predicting death and survival without neurodevelopmental impairment. These models also had similar calibration as measured by the average predicted probabilities and by calibration intercepts and slopes. However, the sum of all predicted probabilities from the logistic models for each infant ranged from 87.7–124.0%. We found that the logistic model of neurodevelopmental impairment had slightly smaller C-statistics and among infants whose sum of all predicted probabilities did not equal 100% it had worse calibration.

To overcome potential bias due to death as a competing risk, we applied an extension of logistic regression method, logistic competing risks regression, to develop a prediction model of neurodevelopmental impairment. Because time to diagnosis of NDI was determined only at one fixed time, 22–26 months’ age corrected for prematurity, the estimated odds ratios for predictor variables were close to those in the logistic model of neurodevelopmental impairment. We also estimated a logistic model of composite of neurodevelopmental impairment or death and showed that it could not be used for predicting neurodevelopmental impairment. Competing risks are not only of statistical interest, but also can be of substantive interest. In pediatric research, for example, it is increasingly concerned how the risk and burden of illness among extremely preterm infants are changing with improved survival [19]. Further investigation into statistical methods for modeling competing risks and collection of more detailed data on event time will be needed.

Constraining sum of all predicted probabilities of outcome categories for each patient to 100% and accommodating competing risks are important considerations in the validation of prediction models for multicategory outcomes. Additionally, there are other statistical and practical issues that should be considered. We prepared a list of these issues for comparison between dichotomized logistic and multinomial logit regression in Table 4. In general, simplicity in model interpretation facilitates acceptance and usage of a model by clinicians. Flexibility in model fitting to allow outcome category-specific predictor variables helps avoid statistical overfitting and including random-effect terms to accommodate patient heterogeneity by hospital improves model calibration [3].

Both logistic regression and logistic competing risks regression produce odds ratio estimates for predictor variables but have the flaw that sum of all predicted probabilities of outcome categories for each patient is not constrained to 100%. Logistic regression also has the advantages of allowing for outcome-category specific predictor variables and random-effect terms, and wide availability of statistical programs for model estimation. Logistic competing risks regression accounts for competing risks but does not allow the inclusion of random-effect terms for patient heterogeneity and requires time-to-event data.

Multinomial logit regression constrains sum of all predicted probabilities of outcome categories for each patient to 100%. But a standard multinomial logit model has some known limitations, including difficulty to explain the prediction results to clinicians or patients due to the use of a fixed reference category, lack of flexibility to allow for outcome category-specific predictors and complications caused by the inclusion of random-effect terms. As an alternative for ordinal outcome, we estimated a continuous-ratio logit model to predict the probability of death and the probability of neurodevelopmental impairment conditional on surviving. This addressed the need of clinicians and patients for separate information on death and impairment, which could be valued differently in their decision about treatment options. It also afforded us the statistical benefits of including random-effect terms and outcome category-specific predictor variables of neurodevelopmental impairment in the model. The infant outcomes in our data have been found to vary significantly across hospitals after controlling for infant characteristics [15]. To improve the modest model performance in predicting neurodevelopmental impairment, we hope to be able to add more variables predictive of this outcome in the future [20, 21].

## Conclusion

A multicategory outcome is often dichotomized and modeled using logistic regression in studies developing prediction models. Because a single outcome category is often of interest, the shortcomings of this method have not received much attention. Although logistic models and multinomial logit models may have similar predictive performance, logistic models do not constrain predicted probabilities of all outcome categories to 100% for a patient and can produce poorly calibrated predictions. We recommend the use of various alternative forms of multinomial logit models such as continuation-ratio logit models for ordinal outcomes, which allow for the accommodation of patient heterogeneity by hospital and the inclusion of outcome category-specific predictors. To overcome competing-risk bias among outcome categories, modeling composite of outcome categories can lead to misleading predictions. Application of logistic competing risks regression and collection of time-to-event data needed should be explored in future studies.

## Figures and Tables

**Table 1:** Estimated odds ratio (95% CI) and variance (SE) of random hospital effect from logistic models of dichotomized outcomes and continuation-ratio logit model

	Logistic models of dichotomized outcomes			Continuation-ratio logit model	
Predictor variable	Death	NDI	NDI-free Survival	Death	NDI among surviving infants
Birth weight (100 grams)	0.66 (0.62–0.71)	1.05 (0.98–1.12)	1.45 (1.36–1.56)	0.66 (0.62–0.71)	0.80 (0.74–0.87)
Gestational age (weeks)					
22–23	2.76 (2.22–3.43)	0.61 (0.48–0.78)	0.43 (0.33–0.55)	2.59 (2.09–3.22)	1.23 (0.91–1.68)
24	1.46 (1.23–1.72)	0.98 (0.83–1.16)	0.72 (0.61–0.85)	1.46 (1.24–1.72)	1.20 (0.99–1.46)
25	1.00	1.00	1.00	1.00	1.00
Female	0.57 (0.50–0.66)	0.97 (0.84–1.12)	1.85 (1.60–2.15)	0.58 (0.50–0.67)	0.66 (0.55–0.78)
Singleton birth	0.86 (0.74–1.01)	1.05 (0.89–1.24)	1.13 (0.95–1.34)	0.85 (0.73–1.00)	0.97 (0.79–1.18)
Antenatal corticosteroids	0.53 (0.43–0.65)	1.28 (1.03–1.60)	1.73 (1.35–2.21)	0.52 (0.42–0.63)	0.65 (0.49–0.87)
Hospital variance	0.165 (0.065)	0.126 (0.051)	0.294 (0.110)	0.147 (0.055)	0.213 (0.064)

**Table 2:** Odds ratio (95% CI) from models of competing-risk outcome categories

	Logistic competing risk model	Logistic model of composite outcome
Predictor variable	NDI(ref: Death or NDI-free)	NDI or Death (ref: NDI-free)
Birth weight (100 grams)	1.06 (0.98–1.15)	0.69 (0.64–0.74)
Gestational age (weeks)		
22–23	0.58 (0.43–0.78)	2.34 (1.81–3.02)
24	0.98 (0.80–1.20)	1.38 (1.17–1.64)
25	1.00	1.00
Female	0.96 (0.80–1.16)	0.54 (0.47–0.63)
Singleton birth	1.03 (0.83–1.29)	0.89 (0.75–1.05)
Antenatal corticosteroids	1.21 (0.93–1.59)	0.58 (0.45–0.74)

**Table 3:** Measures of model predictive performance

Outcome category	Dichotomized logistic models	Continuation-ratio logit model
	Overall validity: Brier score/corrected on 200 bootstrap samples	
Death	0.199/0.203	0.199/0.202
NDI	0.194/0.196	0.192/0.194
NDI-free survival	0.186/0.189	0.186/0.188
	Discrimination: C-statistics (95% CI)/corrected on 200 bootstrap samples	
Death	0.738 (0.722–0.754)/0.729	0.738 (0.722–0.753)/0.729
NDI	0.623 (0.604–0.643)/0.606	0.637 (0.618–0.656)/0.619
NDI-free survival	0.730 (0.714–0.746)/0.720	0.730 (0.713–0.746)/0.721
	Calibration: mean of predicted probability (range)	
Death	40.3 (3.7–91.6)	40.3 (4.0–91.2)
NDI	28.1 (8.5–48.8)	28.1 (6.6–52.1)
NDI-free survival	31.5 (1.5–81.0)	31.6 (0.8–78.9)
Sum over all categories	100.0 (87.7–124.0)	100.0 (100.0–100.0)
	Calibration intercept/slope	
Death	0.009/1.026	0.020/1.051
NDI	0.121/1.133	0.164/1.184
NDI-free survival	0.023/1.035	0.045/1.076

**Table 4:** Comparison of predictive modeling methods on other statistical and practical issues

	Methods for risk prediction of multicategory outcomes		
Issues to consider	Dichotomized logistic regression	Continuation-ratio logit regression	Logistic competing risks regression
Interpretation of predictor effects	Odds ratio	Conditional odds ratio dependent on ordered outcome category	Odds ratio
Constrains sum of all predicted probabilities to 100%	No	Yes	No
Allows inclusion of random-effect terms	Yes	Yes	No/Robust variance estimates
Allows inclusion of outcome category-specific predictor variables	Yes	Yes	Yes
Accommodates competing risks	No	Yes	Yes
Availability in statistical software	SAS, Stata, R	SAS, Stata, R	R

## Data Availability

The datasets used are available from Dr. Lei Li on reasonable request.
